# Impedimetric Label-Free Immunosensor on Disposable Modified Screen-Printed Electrodes for Ochratoxin A

**DOI:** 10.3390/bios6030033

**Published:** 2016-06-30

**Authors:** Francesca Malvano, Donatella Albanese, Alessio Crescitelli, Roberto Pilloton, Emanuela Esposito

**Affiliations:** 1 Department of Industrial Engineering, University of Salerno, 84084 Fisciano SA, Italy; fmalvano@unisa.it; 2 Institute for Microelectronics and Microsystems of the National Council of Research (CNR), 80131 Napoli, Italy; alessio.crescitelli@cnr.it (A.C.); emanuela.esposito@cnr.it (E.E.); 3 Institute of Atmospheric Pollution Research of the National Council of Research (CNR), 00015 Roma, Italy; roberto.pilloton@cnr.it

**Keywords:** immunosensor, screen printed electrodes, Ochratoxin A, electrochemical impedance spectroscopy

## Abstract

An impedimetric label-free immunosensor on disposable screen-printed carbon electrodes (SPCE) for quantitative determination of Ochratoxin A (OTA) has been developed. After modification of the SPCE surface with gold nanoparticles (AuNPs), the anti-OTA was immobilized on the working electrode through a cysteamine layer. After each coating step, the modified surfaces were characterized by cyclic voltammetry (CV) and electrochemical impedance spectroscopy (EIS). The capacitance was chosen as the best parameter that describes the reproducible change in electrical properties of the electrode surface at different OTA concentrations and it was used to investigate the analytical parameters of the developed immunosensor. Under optimized conditions, the immunosensor showed a linear relationship between 0.3 and 20 ng/mL with a low detection limit of 0.25 ng/mL, making it suitable to control OTA content in many common food products. Lastly, the immunosensor was used to measure OTA in red wine samples and the results were compared with those registered with a competitive ELISA kit. The immunosensor was sensitive to OTA lower than 2 μg/kg, which represents the lower acceptable limit of OTA established by European legislation for common food products.

## 1. Introduction

Mycotoxins are a large and varied group of mold-secondary metabolites with common features because they are all produced by fungi and have toxic effects against vertebrates and other organisms. Mycotoxins affect a broad range of agricultural products, including cereals, cereal-based foods, dried fruits, wine, milk, coffee beans, cocoa, or meat products, which are the basis of the economies of many developing countries [1]. Moreover, mycotoxins are presently considered as the most important chronic dietary risk factor, higher than food additives or pesticide residues [2].

Ochratoxin A (OTA) is one of the most abundant mycotoxins that contaminates food products; it is found in tissue and organs of animals, including human blood and breast milk, and is known to produce nephrotoxic, teratogenic, carcinogenic, and immune toxic activity in several animal species [3]. OTA affects humans mainly through consumption of improperly stored food products, causing cancer. 

The International Agency of Research on Cancer (IARC) has classified OTA as a possible carcinogenic compound for humans since it causes immune suppression and immune toxicity [4].

From the mid-2000s, approximately 100 countries (covering 85% of the world’s inhabitants) had specific regulations or detailed guidelines for the occurrence of mycotoxins in food [2]. The European Union has established with the Regulation (EC) No 1881/2006, stating the acceptable limits for OTA in many products at high risk of contamination: OTA is allowed in very small concentrations (about 0.5–10 μg/Kg) depending on the kind of food [5]. These regulatory limits force all Member States to monitor and control mycotoxin levels in foodstuffs in order to reduce the intake of this toxic compound.

The methods most frequently used for OTA determination are thin-layer chromatography (TLC), high-performance liquid chromatography (HPLC), coupled to fluorescence or mass spectrometry detectors [6]. The chromatographic techniques are highly sensitive and specific, but require well-equipped laboratory facilities, time-consuming pretreatment steps, and highly-trained personnel that strongly limit the routine implementation of chromatography-based approaches [7]. Immune assays can be used as cheaper and quicker alternatives to chromatographic methods for mycotoxin detection. Competitive enzyme-linked immune sorbent assay (ELISA) is the most common commercial immune assay used for OTA detection in the food sector based on spectrophotometric reading, however, it suffers the drawbacks of time-consuming (for example, 50 min for I’screen Ochra Elisa kit (Tecna-Trieste, Italy); 90 min for OTA ELISA, (Abraxis LLC, Warminster, PA, USA), and the narrow dynamic range up to 16 ng/mL. 

In recent years there has been a strong interest in the development of immunosensors based on the antigen-antibody interaction, but novel specific ligands (e.g., aptamers) are emerging.

Among all of the possible immunosensors (electrochemical, optical, microgravimetric) the electrochemical ones rank highly owing to their sensitivity, low cost, simplicity and, in some cases, miniaturization, portability, and integration in automated devices [8].

In the literature are reported two different types of electrochemical OTA immunosensors: label- and label-free-based. For the labelled immunosensors, the interaction between OTA and anti-OTA, based on direct and/or indirect competitive immunoassay, is detected by the use of enzymes exploiting the classical electrochemical techniques, such as amperometric, potentiometric, and conductimetric methods [9,10,11,12,13].

This immunosensor exhibits a detection limit ranging from 0.008 [12] to 0.12 [10] ng/mL with a dynamic range up to 250 ng·mL [13].

The application of electrochemical impedance spectroscopy (EIS), as a transduction technology, enables the label-free detection and quantification of the immune complex and, thus, for the development of biosensors for food hazards [14,15]. EIS is a powerful informative and non-destructive technique due to the small voltage excitation used during detection, which can be used to study the electrical properties of the sensing device interface and tracing the reactions occurring on it [16,17]. 

Different impedimetric label-free OTA affinity biosensors have been described previously by immobilization of monoclonal antibody and aptamers on the surface of gold or platinum electrodes [18,19,20,21]. This impedimetric immunosensor exhibits detection limit ranging from 0.01 [19] to 2 [20] ng/mL, with and a dynamic range up to 25 ng/mL [21].

In recent years the applications of disposable screen-printed carbon electrodes (SPCEs), characterized by low-cost fabrication and mass production, have attracted an increasing interest for the development of labelled immunosensors (especially enzyme immunosensors) but it is noteworthy that few studies [22,23] on electrochemical label-free immunosensors integrated onto SPCEs have been developed.

Exploiting the advantages of gold nanoparticles (AuNPs), which have been extensively used as matrices for the immobilization of macromolecules, such as proteins, enzymes, and antibodies, in addition to providing a microenvironment similar to what obtained under physiological conditions [24], it is possible to design a new electrochemical sensor by the modification of a working electrode surface. Moreover, AuNPs have attracted considerable attention in electroanalysis because of their excellent physical and chemical properties, such as high surface to volume ratio, good electrical properties, strong adsorption ability, and good surface properties. Electrodeposition of metallic nanoparticles on an electrode surface is a better process than deposition from solution, as the former is comparatively easier, faster, and generates a more stable surface. In addition, electrochemical deposition allows Au(III) to be addressed only on a polarised working electrode as Au(0) not on auxiliary or reference electrodes. Compared to gold electrodes, the electrodeposition of metallic nanoparticles or aggregated clusters (simply abbreviated as nanoparticles in this paper) on SPEs is more advantageous because of the unique properties offered by the metallic nanoparticles as described earlier [25]. Moreover the gold deposition on the electrode avoids poisoning and cross-contamination, which interferes with the analysis. These problems are absent in disposable SPCEs. Consequently, the combination of screen-printing and electrodeposition of metallic nanoparticles is a very promising technique for the mass production of electrochemical sensors with enhanced sensitivity [25]. 

For this reason, the aim of this work is the development of a label-free impedimetric immunosensor for OTA detection, realized on an AuNP-modified SPCE. EIS and CV were used to characterize each step of electrode modification and the analytical performances of the immunosensors developed. Finally the possibility to use a fast and cheap disposable biosensor like that proposed in this study could represent a key factor for the monitoring of mycotoxins in food products.

## 2. Materials and Methods 

### 2.1. Chemicals

Glutaraldehyde solution (C_5_H_8_O_2_, 50 wt % in H20), cysteamine (C_2_H_7_NS, ≤98%), gold (III) chloride hydrate (HAuCl_4_, 99.9%), sulfuric Acid (H_2_SO_4_, 99.9%), ethanolamine (NH_2_CH_2_CH_2_OH, >99.5%), potassium hexacyanoferrate (III) ([Fe(CN)_6_]^3−^, >99%), and Ochratoxin A were purchased from Sigma-Aldrich (Milano, Italy). Potassium ferrocyanide ([Fe(CN)_6_]^4−^) was obtained from Carlo Erba reagent (Milano, Italy). Anti-Ochratoxin A antibody (Anti OTA, 1 mg/mL) was purchased from Abcam (Cambridge, UK), NaH_2_PO_4_, Na_2_HPO_4_, NaCl, and KCl used in the preparation of phosphate-buffered saline (PBS: 0.1 M KCl, pH 7.4) were also obtained from Sigma Aldrich (Milano, Italy). I’screen Ochra ELISA kit for the detection of Ochratoxin A was purchased from Tecna (Italy).

### 2.2. Apparatus

**Figure 1:**
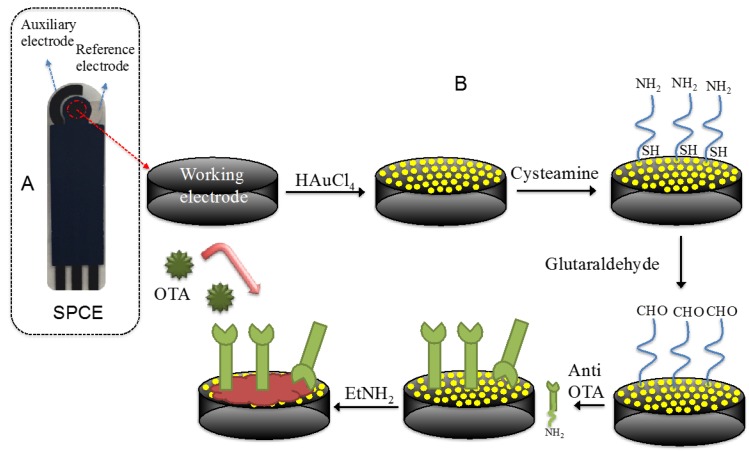
(**A**) Screen-printed carbon electrode layout; and (**B**) steps used for the fabrication of the immunosensor.

The electrochemical measurements were carried out with a computer-controlled Autolab PGSTAT 204 Potentiostat (Metrohm), equipped with an impedance module (FRA32M) and the experimental data were analysed with Nova software (Metrohm). Screen-printed carbon electrodes (SPCEs), based on a three-electrode layout (working/auxiliary/reference; Figure 1A), were produced in three screen-printing steps as described in Albanese et al. [26]. Specifically, a first layer of a carbon/graphite ink (G-Went, Pontypool, UK) was deposed to define the conducting tracks, the working and auxiliary electrodes. The second was a silver/silver chloride ink (Acheson Colloiden B.V., Scheemda, The Netherlands), used as a pseudo-reference electrode. The third layer consisted in an insulating ink (G-Went, Pontypool, UK). Between the first and the second screen-printing steps, the strips were cured at 80 °C for 25 min to dry off residual solvents and cure the patterned pastes after every step of screen-printing. The diameter of the working electrode was 2.8 mm. Scanning electron microscopy (SEM) images were obtained on a Raith Turnkey 150 SEM. 

### 2.3. Immunosensor Manufactoring

#### 2.3.1. Preparation of Gold-Modified SPCEs 

The gold deposition on the homemade SPCEs was conducted after an electrochemical treatment at 1.7 V vs. Ag/AgCl as a reference electrode for 360 s in PBS. 

The electrochemical AuNP deposition was carried out using a solution of 1 M HAuCl_4_ in 0.5 M H_2_SO_4_ under a constant potential of −0.4 V vs. Ag/AgCl in a range of 50–400 s.

#### 2.3.2. Electrochemical Deposed Multilayer (EDM)

EDM was employed to create thiol layers attached onto AuNPs. Cysteamine 20 mM was dropped onto the AuNPs modified working electrode and a constant potential of 1.2 V vs. Ag/AgCl for 20 min was applied. After the electrode was thoroughly rinsed with water, to remove physically-adsorbed cysteamine, 100 μL of glutaraldehyde solution 12% (v/v) were dropped onto the modified working electrode for 1 h and then, again, rinsed with water.

#### 2.3.3. Antibody Immobilization

Different concentrations of anti-OTA solution (1 μg/mL, 5 μg/mL, 10 μg/mL) were dropped onto the modified electrode for 30 min at room temperature, then the electrode was rinsed in PBS to remove unbound antibodies. After the immobilization step, ethanolamine 1 M (pH 8.5) for 15 min was used to block unreacted active sites. The schematic diagram of immunosensor fabrication is shown in Figure 1B. 

### 2.4. Experimental Measurement

EIS and CV measurements were used to characterize each step of the electrode modification.

For the impedance measurements, a sinusoidal AC potential (10 mV) in the frequency range from 0.1 to 10^4^ Hz was superimposed to 0.00 mV (vs. reference electrode) DC potential. The impedance spectra were plotted in the form of Nyquist plots, where the complex impedance is displayed as the sum of the real and imaginary components (Z’ and Z’’ respectively), and in the form of Bode diagram where the total impedance of the system (Z) is plotted versus frequency. All measurements were performed in a solution of 1 mM ferri/ferrocyanide redox couple ([Fe(CN)_6_]^4−^/^3−^, 1:1) in PBS, pH 7.5, as background electrolyte at room temperature. 

The voltammetric measurements were performed from −0.6 to 0.6 V vs. Ag/AgCl with a scan rate of 0.05 V/s; the redox couple used for the CV was the same as that used for impedance measurements*.*

For the OTA analysis, 20 μL of OTA at different concentrations in PBS were dropped onto the working area of the immunosensor and incubated for 20 min. Before the impedance measurements, the immunosensor was rinsed thoroughly with copious amounts of PBS.

### 2.5. Preparation of Wine Samples for OTA Measurement

The preparation of red wine samples was conducted according to the procedure described in the I’screen Ochra ELISA kit. 5 mL of wine was added to 5 mL of 1 M HCl and 10 mL of dichloromethane; the solution was shaken in a low-speed shaker (400 rpm) for 15 min and centrifuged at 2200 rpm for 15 min. Five millilitres of solvent phase was added to 2.5 mL of the 0.13 M sodium bicarbonate solution; the solution was shaken for 30 s. The upper aqueous phase was recovered and centrifuged to remove solvent traces and finally diluted two times with sodium bicarbonate solution. 

This procedure was replicated for the three different OTA (1.5 ng/mL, 5 ng/mL, and 10 ng/mL) spiked wine samples. The OTA results obtained by the immunosensors were compared with those measured with the competitive ELISA kit for OTA.

## 3. Results

### 3.1. Characterization of the Electrode Modifying Process

**Figure 2:**
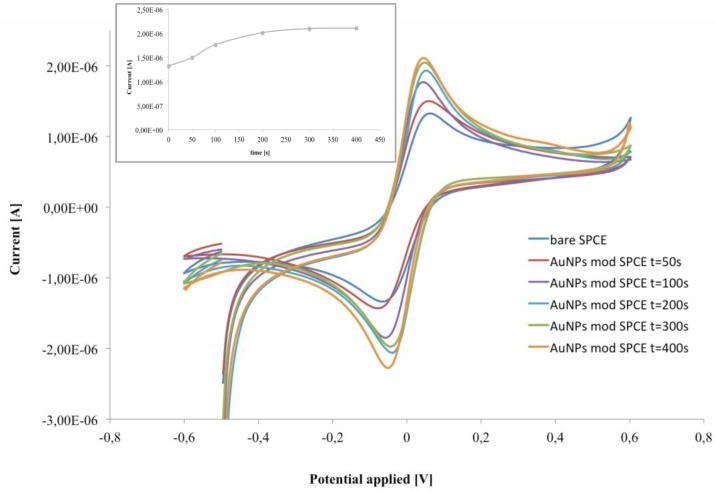
CV of bare and AuNP-modified SPCE at different deposition times. Inset: current intensity of the anodic peak at different deposition times, in 1 mM ferri/ferrocyanide redox couple ([Fe(CN)_6_]^4−/3−^, 1:1) in PB, pH 6.8.

The electrodeposition of AuNPs on the electrode surface was strongly affected by the electrodeposition time, which have been optimized to obtain the best analytical performances in our device. As Figure 2 shows, an increase of the electrodeposition time from 50 to 300 s leads to significant current increase, while an effect that plateaued at a longer deposition time is observed (inset Figure 2). Therefore, an electrodeposition time of 300 s was selected as optimum. 

As expected, the working electrode modified with AuNPs exhibited the characteristic increase of the anodic and cathodic peaks, thus confirming the successful modification process. Moreover increasing the electrodeposition time from 50 to 300 s, larger peak currents and a smaller peak-to-peak potential separations (ΔE) were observed. In particular, the optimized AuNP deposition time increased the electrochemical performance of the electrode with anodic current of 2.1 μA and ΔE = 91 mV for AuNPs/SPCE, in contrast to 1.3 μA and ΔE = 135 mV for SPCE (Figure 2). This behaviour was attributed to the enhanced electrochemical activity of the AuNPs, which allowed the increase of the electrode active area and shifted the peak potential near to 0 V giving rise to a smaller peak-to-peak separation.

This fact suggests a slight improvement in the electrocatalytic properties of the electrode produced by the addition of the AuNPs, which facilitated the electron-transfer process [27]. 

**Figure 3:**
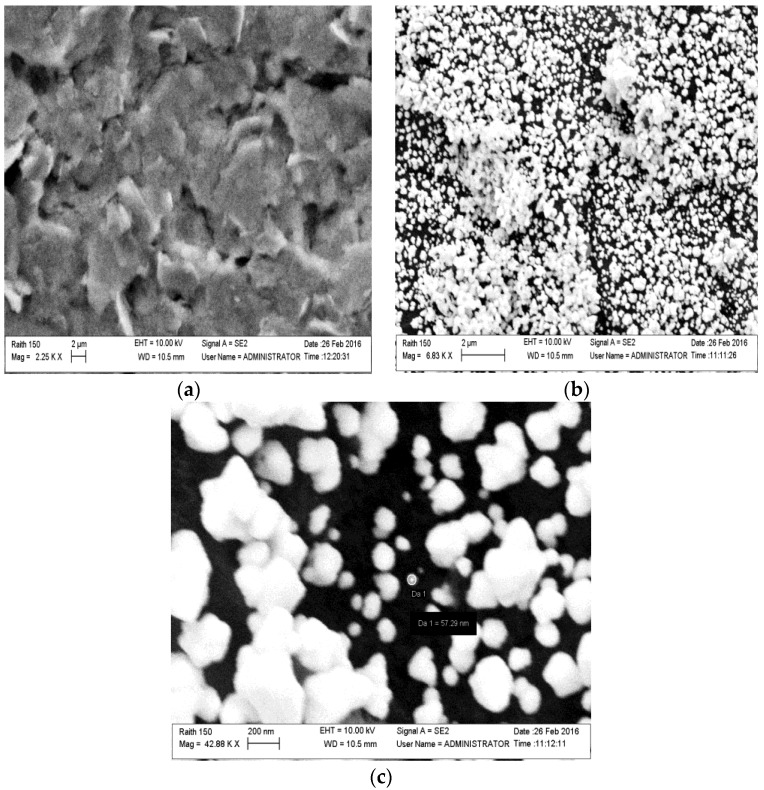
SEM surface images of (**a**) a bare carbon electrode; and (**b**,**c**) a carbon electrode after gold electrodeposition.

SEM images (Figure 3) of the carbon working electrode before and after the gold electrodeposition had been carried out, in order to verify the presence of the AuNPs. As expected, the surface of the bare carbon electrode shows the typical flake-shaped graphite particles along with large cavities. When the electrode is biased to deposit (reduce) the gold, the tips, the reliefs, and depressions of the SPCE surface suffer from a heterogeneous distribution of electric charge that promotes the formation of a heterogeneous dimension of Au particles, besides considering that a high time of deposition favours the interfusion of NPs in larger gold ones so the nano-structure was destroyed. Cluster agglomeration of AuNPs and single NPs are evident in Figure 3b,c. Moreover the quantity of deposed gold on carbon printed surface (122 pg per electrode) has been calculated from the deposition current (0.6 μA) and time (300 s), considering the Faraday constant = 96,485.34 C/mol, the number of electrons (3) involved in the reduction of Au(III) to Au(0), and the atomic weight of Au (196.96 D). AuNPs deposed on SPCE resulted in a cheap gold surface since it was obtained with a smaller quantity of gold than the expensive commercial solid gold electrodes.

**Figure 4:**
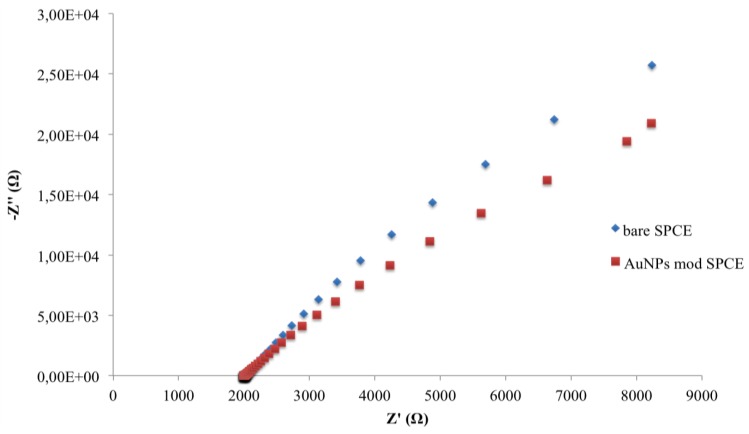
Nyquist plots in impedance measurements of bare and AuNP-modified SPCEs.

Experimental complex plane impedance spectra for the bare SPCE and SPCE-AuNPs are shown in Figure 4. The almost linear and close-to-vertical spectra, observed for both electrodes, is caused by a faster mass-transfer limited process due to electron flow from the electrode surface in the bulk solution. This behaviour indicates a purely capacitive response of the electrode properties [28]. When the AuNPs are deposed, a decrease of curve slope was observed.

**Figure 5:**
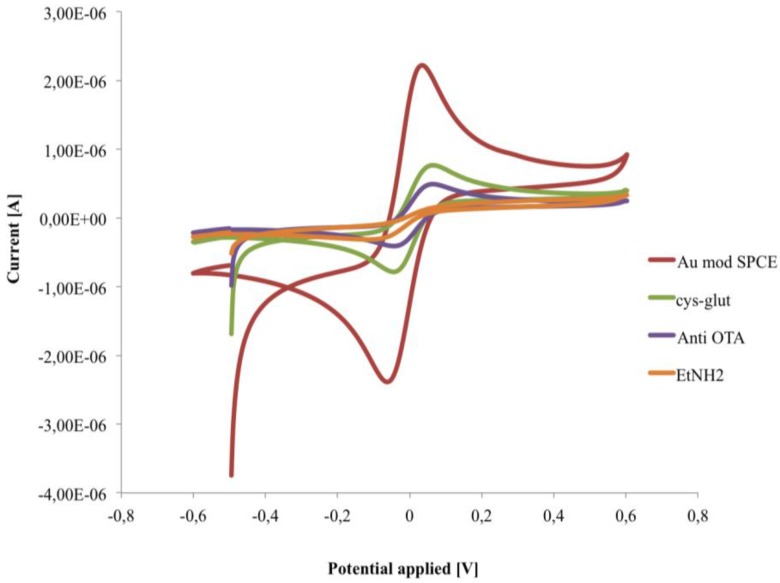
Cyclic voltammograms recorded in 1 mM [Fe(CN)_6_]^4−^/^3−^, in PB pH 6.8, during the fabrication of the immunosensor.

**Figure 6:**
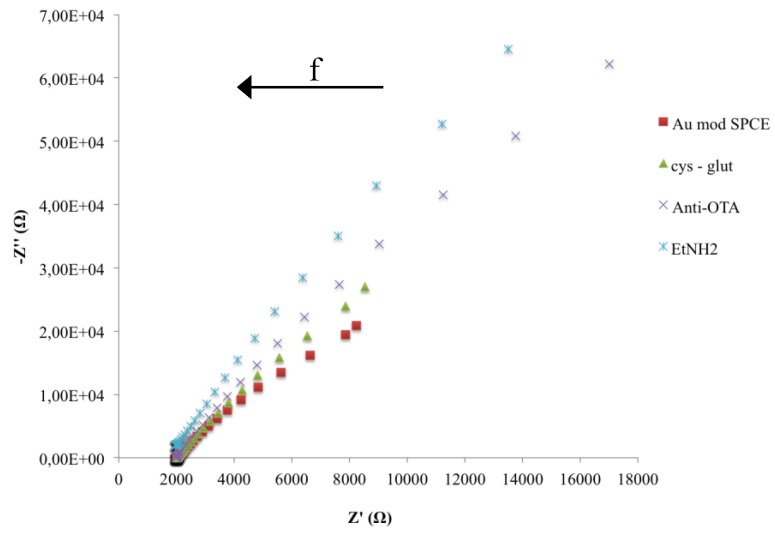
Nyquist plots in impedance measurements during the fabrication of the immunosensor.

In order to characterize the behaviour of the sensing layers, EIS and CV measurements have been carried out during each step of the immunosensor construction (Figure 5 and Figure 6). 

The cysteamine layers, as well as the consecutive immobilization of anti-OTA and ethanolamine, caused a significant decrease of the electron transfer rate, measured by CV, with a lowering of both anodic and cathodic peaks due to hindering effects of the layers [29,30] (Figure 5). 

Impedance spectra, during the immunosensor fabrication, represented as the change of the two impedance components (Z’ resistive component, Z’’ capacitive component), as a function of frequency, were shown in Figure 6. The increase of both *Z* components is due to a change in the electron-transfer resistance caused by the biocomposite layer on the surface of the electrode that also induces a capacitance decrease because of the increased distance in the plate separation between the surface of the electrode and electrolyte solution. Moreover it is evident that a significant change of impedance components occurs only at low frequencies. 

**Figure 7:**
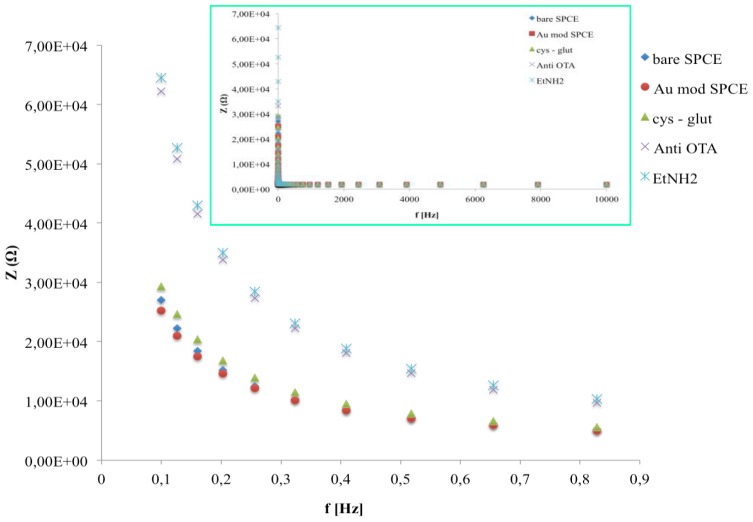
Bode plots in impedance measurements after all immunosensor fabrication steps in the frequency range 0.1–1 Hz. The inset shows Bode plots in the frequency range 0.1–10,000 Hz.

For a better description of the change caused by the immobilization steps on the impedance properties of the immunosensor, the Bode plot (total impedance in function of frequency) have been reported (Figure 7). While no differences were shown at the higher frequency region (inset of Figure 7) significant total impedance changes were shown from 0.1 to 1 Hz. 

In this range no significant differences were observed when the cysteamine layer was attached to AuNPs, in contrast to the immobilization of anti-OTA molecules, which gives rise to a substantial total impedance increase. No changes in impedance value were observed after the blocking of active sites with EtNH_2_. 

**Figure 8:**
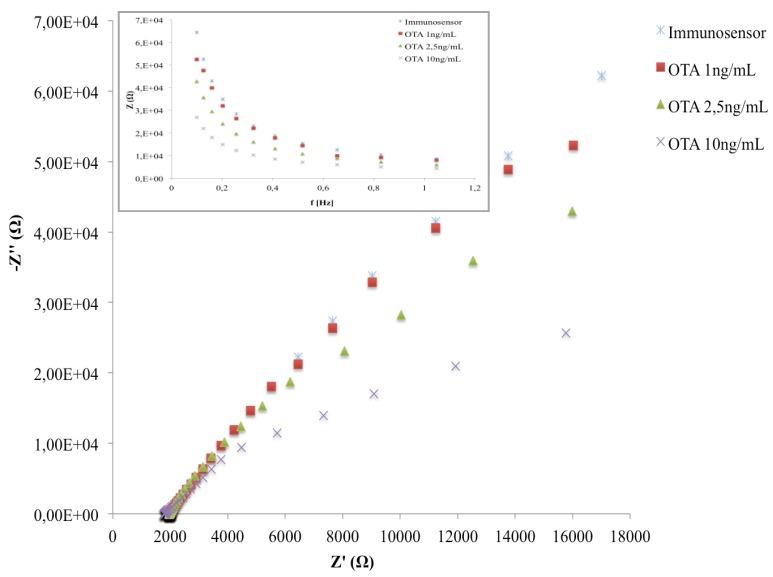
Nyquist plot in impedance measurements of the immunosensor before and after the interaction with different OTA concentrations.

The Nyquist plots of the developed immunosensor after the incubation with three different OTA concentrations are reported in Figure 8. In the given frequency range, the binding of OTA with anti-OTA affects the sensor impedance signal; in particular, we observe a decrease in the capacitive component (–Z’’) of total impedance at low frequencies. According to other studies [31,32,33,34], on impedimetric immunosensors, the making of the immunocomplex induces a capacitance decrease, which can be directly related to the amount of analyte to be quantified.

As shown in the inset of Figure 8, the maximum differences among the Bode plots corresponding to different OTA amounts were observed at 0.1 Hz. The latter was chosen as the operating frequency for all impedance measurements during the analytical performances of the immunosensor. 

### 3.2. Optimization of Anti-OTA Concentration.

The influence of the antibody concentration on the immunosensor analytical performance was investigated. For this reason, immunosensors were developed by the immobilization of three different amounts of anti-OTA (1 μg/mL, 5 μg/mL, 10 μg/mL) and the capacitance (C) was measured for OTA from 0.3 ng/mL to 40 ng/mL, after an incubation time of 20 min*.* The capacitance of the system was calculated according to Yang et al. [33], using the following equation:

(1) $$C=-\frac{1}{2\pi fZ\prime \prime }$$

where f is the operating frequency (Hz), and *Z*” is the capacitive component of the total impedance.

Throughout the whole study, the change in capacitance (denoted as Δ*C*), taken as a measure before and after immunoreaction, is calculated by the following equation:

(2) $$\Delta C=C_{anti\;OTA-OTA}-C_{anti\;OTA}$$

where $C_{anti\;OTA-OTA}$ is the value of the capacitance after OTA coupling to the anti-OTA and $C_{anti\;OTA}$ represents the value of the capacitance of the native immunosensor. 

**Figure 9:**
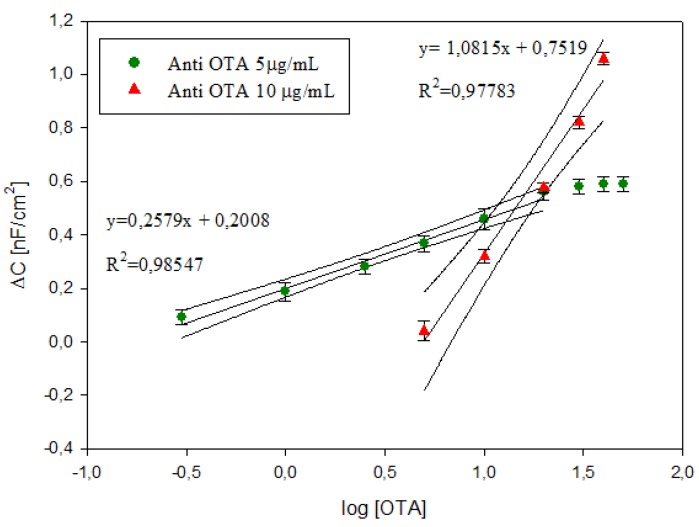
Calibration curves of OTA immunosensors at 5 μg/mL and 10 μg/mL anti-OTA. Data represent the average values of five immunosensors with error bars and 95% confidence curves. The inset shows the measured capacitance in the range of OTA investigated for the immunosensors with 5 μg/mL and 10 μg/mL anti-OTA.

For the immunosensor with 1 μg/mL anti-OTA no significant changes in capacitance was measured before and after the immunocomplex in the range of OTA investigated. The calibration curves of the OTA immunosensors with 5 μg/mL and 10 μg/mL anti-OTA, obtained by plotting the logarithmic value of OTA concentrations versus Δ*C*, are shown in Figure 9, As reported in our previous study [29], lower antibody amounts allow detecting lower OTA concentrations; in particular, for 5 μg/mL anti-OTA, the immunosensor shows a significant Δ*C* in the range from −0.52 to 1.30 log OTA (0.3 to 20 ng/mL), while Δ*C* changes only for values higher than 0.69 log OTA (5 ng/mL) with 10 μg/mL anti-OTA. Moreover, higher antibody amounts allow obtaining higher sensitivity and a higher capacitance signal (see inset Figure 9) due to the higher antigen-binding capacity. 

The detection limit (LOD), calculated using the sum of average blank solution and three times the standard deviation, was 0.37 ng/mL and 5.42 ng/mL for immunosensor with 5 μg/mL and 10 μg/mL, respectively. The comparison of the analytical performance of the label-free immunosensors on screen-printed AuNP-modified carbon electrodes developed in this study with the other impedimetric immunosensor reported in the literature is reported in Table 1. Since no previous studies have been published on the label-free immunosensor on screen-printed carbon electrodes which shows capacitive behaviour, we have compared the results with resistive immunosensors developed on Au and Pt electrodes. In the Table 1 the sensitivity of our immunosensors has been calculated using the change in total impedance as a function of different OTA amounts (data not reported). The analytical parameters obtained by our label-free impedimetric immunosensors are suitable for the analytical determination of OTA in the range of interest for food matrices. In addition, considering that the electrodes used for the current fabrication of impedimetric immunosensors are pure gold electrodes (e.g., thin-film gold electrodes require 2270 pg of gold) our impedimetric immunosensors are cheaper, because of the use of disposable SPCE modified with a very small quantity of gold (calculated to be 122 pg of gold per electrode).

The reproducibility calculated on five different immunsensors showed a good relative standard deviation (RSD) for both immunosensors: 5.18% for 5 μg/mL anti-OTA and 2.5 ng/mL OTA, 5.69% for 10 μg/mL anti-OTA and 20 ng/mL OTA. The feasibility of applying the proposed immunosensor for the detection of OTA in wine was studied. Since the maximum OTA concentration permitted by European Commission (EC) No 1881/2006 is 2 μg/kg we have tested the immunosensor at 5 μg/mL anti-OTA. Finally, the storage stability was also determined. For this purpose different immunosensors were stored for three weeks at 4 °C without chemical preservatives and characterized at regular interval times. After the investigated storage period, the immunosensors showed a negligible loss of activity.

Red wine samples were spiked with three different concentrations of OTA, and analysed by the developed immunosensors and a competitive ELISA kit for OTA detection. The sample preparation used was the same for both the analytical methods. The results, reported in Table 2, show a comparable performance for both methods and, thus, the capability of the immunosensor is a fast analytical technique for the control of OTA in food samples. 

## 4. Conclusions 

The first immunosensor for OTA detection based on EIS with a modified SPCE is reported. The surface of ca arbon electrode was modified with electrochemical gold deposition, which has demonstred a very cheap way to obtain gold-like behaving electrodes using a very small quantity of the metal (122 pg per electrode). Thus, a label-free impedimetric immunosensor for Ochratoxin A detection was developed on Au modified SPCE and EIS was used to analyze the analytical immunosensor performance. Capacitance was chosen as the best parameter that describes the electrical changes of the electrode surface due to the immunoreaction between anti-OTA and OTA at different concentrations. The developed immunosensor, with its very low detection limit and high sensitivity, exploits the advantages of cheapness, simplicity, and versatility of the SPCE and its results are suitable for fast OTA measurement in food matrices.

## Figures and Tables

**Table 1:** Comparison among impedimetric OTA label-free biosensors.

Schematic Immunosensor Assembly	Sensitivity (kΩ mL/ng)	Linear Range (ng/mL)	LOD (ng/mL)	Sensitivity x LOD	References
Pt/PANI-PV-SO^3−^/Ab	0.56	2–10	2.00	1.12	[20]
Au/TA/GA/BSA/Ab–MNP	6.50	0.05–1	0.01	0.06	[19]
Au/4-CP/Ab	20.25	1–20	0.50	10.12	[18]
Au/MBA/ProtA-G/Ab	14.03	0.010-5	0.010	0.14	[29]
Au/MBA/Ab	377.78	0.005–0.050	0.005	1.89	[29]
SPCE/AuNPs/Cys-Glut/Ab(5 μg/mL)	2.56	0.3–20	0.37	0.64	This work
SPCE/AuNPs/Cys-Glut/Ab(10 μg/mL)	3.09	5–40	5.42	7.72	This work

PANI-PV-SO^3−^: Polyaniline–polyvinylsulfonate; TA: Thiolamine SAM; GA: Glutaraldehyde; MNP: Magnetic Nanoparticles. *4-CP: 4-Carboxyphenyl*; *MPA: 4 Mercaptobenzoic acid*; *Ab: OTA Monoclonal Antibody.*

**Table 2:** OTA results in red wine samples obtained by ELISA and the developed impedimetric immunosensor.

	Spiked Concentration (μg/L)	Found Concentration (μg/L)	Recovery (%)
ELISA	1.50	1.49	99.77
	5.00	6.12	122.45
	10.00	9.90	98.96
Impedimetric immunosensor	1.50	1.36	94.56
	5.00	4.99	99.79
	10.00	10.29	102.91
